# 4-[(2-Methyl-5-oxo-4,5-dihydro-1,3-oxazol-4-yl­idene)meth­yl]phenyl acetate

**DOI:** 10.1107/S1600536809032243

**Published:** 2009-08-22

**Authors:** Pengran Guo, Chang Wang, Jianghan Chen, Dehai Mou

**Affiliations:** a School of Environmental Science and Engineering, Sun Yat-Sen University, Guangzhou 510275, People’s Republic of China; b Guangdong Provincial Key Laboratory of Emergency Testing for Dangerous Chemicals, China National Analytical Center, Guangzhou 510070, People’s Republic of China

## Abstract

In the title compound, C_13_H_11_NO_4_, an intramolecular C—H⋯N interaction helps to establish the conformation. In the crystal, two C—H⋯O contacts stack adjacent mol­ecules into a one-dimensional double chain running in the *a*-axis direction.

## Related literature

The title compound is an important medical inter­mediate, see: Baker (1951 ▶). 
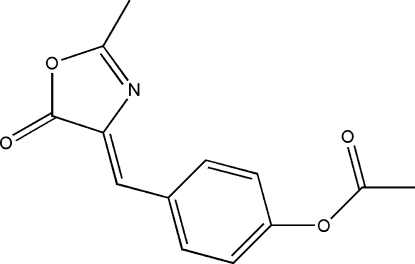


## Experimental

### 

#### Crystal data

C_13_H_11_NO_4_
*M* *_r_* = 245.23
Triclinic, 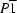

*a* = 5.5802 (15) Å
*b* = 7.446 (2) Å
*c* = 15.012 (4) Å
α = 94.322 (4)°
β = 93.156 (4)°
γ = 108.136 (4)°
*V* = 589.1 (3) Å^3^
*Z* = 2
Mo *K*α radiation
μ = 0.10 mm^−1^
*T* = 293 K
0.14 × 0.13 × 0.08 mm

#### Data collection

Bruker SMART APEX CCD area-detector diffractometer
Absorption correction: multi-scan (*SAINT-Plus*; Bruker, 2003 ▶) *T*_min_ = 0.985, *T*_max_ = 0.992
3324 measured reflections
2264 independent reflections
1707 reflections with *I* > 2σ(*I*)
*R*_int_ = 0.016

#### Refinement

*R*[*F* ^2^ > 2σ(*F* ^2^)] = 0.061
*wR*(*F* ^2^) = 0.164
*S* = 1.04
2264 reflections
153 parameters
H-atom parameters constrained
Δρ_max_ = 0.15 e Å^−3^
Δρ_min_ = −0.14 e Å^−3^

### 

Data collection: *SMART* (Bruker, 1998 ▶); cell refinement: *SAINT-Plus* (Bruker, 1998 ▶); data reduction: *SAINT-Plus*; program(s) used to solve structure: *SHELXS97* (Sheldrick, 2008 ▶); program(s) used to refine structure: *SHELXL97* (Sheldrick, 2008 ▶); molecular graphics: *SHELXTL* (Sheldrick, 2008 ▶); software used to prepare material for publication: *SHELXTL*.

## Supplementary Material

Crystal structure: contains datablocks global, I. DOI: 10.1107/S1600536809032243/kj2130sup1.cif

Structure factors: contains datablocks I. DOI: 10.1107/S1600536809032243/kj2130Isup2.hkl

Additional supplementary materials:  crystallographic information; 3D view; checkCIF report

## Figures and Tables

**Table 1:** Hydrogen-bond geometry (Å, °)

*D*—H⋯*A*	*D*—H	H⋯*A*	*D*⋯*A*	*D*—H⋯*A*
C4—H4⋯N1	0.93	2.45	3.098 (4)	127
C7—H7⋯O2^i^	0.93	2.53	3.417 (4)	160
C13—H13*B*⋯O4^ii^	0.96	2.50	3.382 (4)	152

Symmetry codes: (i) 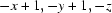; (ii) 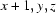.

**Fig. 1.**
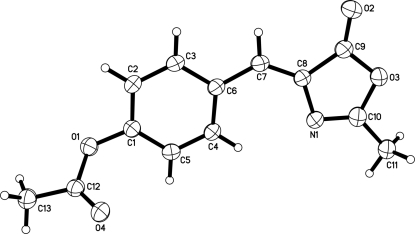
A view of the title compound with the atom-labeling scheme and 30% probability displacement ellipsoids.

**Fig. 2.**
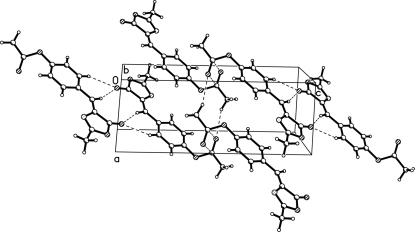
Perspective view along the b-axis of the crystal packing of the title compound. Dashed lines indicate C-H···O and C-H···N contacts.

## supplementary crystallographic information

### Comment

The title compound is an important medical intermediate (Baker, 1951). Here we report the molecular and crystal structure of the title compound (Fig. 1). In the cystal structure,the five- and six-membered rings are nearly coplanar. The interplanar angle between the two rings is 1.392 (3)°. The crystal packing (Fig. 2) is stabilized by two types of intermolecular C—H···O interactions and one kind of intramolecular C—H···N interaction. Details are listed in Table 1. These interactions join the molecules into a double chain parallel to the *a* axis.

### Experimental

The title compund is synthesized according to previous reported literature (Baker, 1951). Single crystals suitable for X-ray diffraction were obtained by slow evaporation of a solution of the title compound in dichloromethane at room temperature.

### Refinement

For Methyls, H atoms were positioned theoretically with *U*_iso_(H) = 1.5*U*_eq_(C). The other H atoms in the title compound were placed geometrically and refined with fixed individual displacement parameters [*U*_iso_(H) = 1.2*U*_eq_(C)], using a riding model.

### Crystal data

**Table:**

C_13_H_11_NO_4_	*Z* = 2
*M**_r_* = 245.23	*F*(000) = 256
Triclinic, *P*1	*D*_x_ = 1.383 Mg m^−^^3^
Hall symbol: -P 1	Mo *K*α radiation, λ = 0.71073 Å
*a* = 5.5802 (15) Å	Cell parameters from 2544 reflections
*b* = 7.446 (2) Å	θ = 3.0–25.4°
*c* = 15.012 (4) Å	µ = 0.10 mm^−^^1^
α = 94.322 (4)°	*T* = 293 K
β = 93.156 (4)°	Block, colourless
γ = 108.136 (4)°	0.14 × 0.13 × 0.08 mm
*V* = 589.1 (3) Å^3^	

### Data collection

**Table:**

Bruker SMART APEX CCD area-detector diffractometer	2264 independent reflections
Radiation source: fine-focus sealed tube	1707 reflections with *I* > 2σ(*I*)
graphite	*R*_int_ = 0.016
Detector resolution: 15 pixels mm^-1^	θ_max_ = 26.1°, θ_min_ = 2.7°
φ and ω scans	*h* = −6→3
Absorption correction: multi-scan (*SAINT-Plus*; Bruker, 2003)	*k* = −9→9
*T*_min_ = 0.985, *T*_max_ = 0.992	*l* = −18→15
3324 measured reflections	

### Refinement

**Table:**

Refinement on *F*^2^	Primary atom site location: structure-invariant direct methods
Least-squares matrix: full	Secondary atom site location: difference Fourier map
*R*[*F*^2^ > 2σ(*F*^2^)] = 0.061	Hydrogen site location: inferred from neighbouring sites
*wR*(*F*^2^) = 0.164	H-atom parameters constrained
*S* = 1.04	*w* = 1/[σ^2^(*F*_o_^2^) + (0.0685*P*)^2^ + 0.3541*P*] where *P* = (*F*_o_^2^ + 2*F*_c_^2^)/3
2264 reflections	(Δ/σ)_max_ < 0.001
153 parameters	Δρ_max_ = 0.15 e Å^−^^3^
0 restraints	Δρ_min_ = −0.14 e Å^−^^3^

### Special details

**Table:**

Geometry. All e.s.d.'s (except the e.s.d. in the dihedral angle between two l.s. planes) are estimated using the full covariance matrix. The cell e.s.d.'s are taken into account individually in the estimation of e.s.d.'s in distances, angles and torsion angles; correlations between e.s.d.'s in cell parameters are only used when they are defined by crystal symmetry. An approximate (isotropic) treatment of cell e.s.d.'s is used for estimating e.s.d.'s involving l.s. planes.
Refinement. Refinement of *F*^2^ against ALL reflections. The weighted *R*-factor *wR* and goodness of fit *S* are based on *F*^2^, conventional *R*-factors *R* are based on *F*, with *F* set to zero for negative *F*^2^. The threshold expression of *F*^2^ > σ(*F*^2^) is used only for calculating *R*-factors(gt) *etc*. and is not relevant to the choice of reflections for refinement. *R*-factors based on *F*^2^ are statistically about twice as large as those based on *F*, and *R*- factors based on ALL data will be even larger.

### Fractional atomic coordinates and isotropic or equivalent isotropic displacement parameters (Å^2^)

**Table:**

	*x*	*y*	*z*	*U*_iso_*/*U*_eq_	
N1	0.4700 (4)	0.9795 (3)	0.17606 (14)	0.0516 (6)	
O1	1.3127 (3)	0.7316 (3)	0.44177 (12)	0.0565 (5)	
O2	0.2844 (4)	0.6674 (3)	−0.02010 (13)	0.0725 (6)	
O3	0.2123 (4)	0.9237 (3)	0.04884 (12)	0.0568 (5)	
O4	1.0311 (4)	0.7157 (3)	0.54417 (13)	0.0631 (6)	
C1	1.1357 (5)	0.7294 (4)	0.37105 (17)	0.0491 (6)	
C2	1.1061 (5)	0.5959 (4)	0.29939 (17)	0.0540 (7)	
H2	1.1950	0.5091	0.3002	0.065*	
C3	0.9434 (5)	0.5922 (4)	0.22626 (17)	0.0518 (7)	
H3	0.9229	0.5019	0.1778	0.062*	
C4	0.8449 (5)	0.8561 (4)	0.29798 (17)	0.0529 (7)	
H4	0.7574	0.9439	0.2980	0.064*	
C5	1.0078 (5)	0.8603 (4)	0.37083 (17)	0.0552 (7)	
H5	1.0312	0.9507	0.4195	0.066*	
C6	0.8087 (5)	0.7219 (3)	0.22381 (16)	0.0460 (6)	
C7	0.6402 (5)	0.7112 (4)	0.14512 (17)	0.0485 (6)	
H7	0.6312	0.6140	0.1010	0.058*	
C8	0.4950 (5)	0.8196 (3)	0.12586 (16)	0.0456 (6)	
C9	0.3284 (5)	0.7834 (4)	0.04328 (18)	0.0517 (6)	
C10	0.3096 (6)	1.0311 (4)	0.1294 (2)	0.0614 (5)	
C11	0.2127 (6)	1.1906 (4)	0.1497 (2)	0.0614 (5)	
H11A	0.2977	1.2619	0.2046	0.092*	
H11B	0.0343	1.1427	0.1558	0.092*	
H11C	0.2430	1.2715	0.1019	0.092*	
C12	1.2360 (5)	0.7171 (4)	0.52634 (17)	0.0483 (6)	
C13	1.4387 (6)	0.6997 (4)	0.5901 (2)	0.0614 (5)	
H13A	1.4521	0.7817	0.6439	0.092*	
H13B	1.5969	0.7353	0.5632	0.092*	
H13C	1.3984	0.5707	0.6044	0.092*	

### Atomic displacement parameters (Å^2^)

**Table:**

	*U*^11^	*U*^22^	*U*^33^	*U*^12^	*U*^13^	*U*^23^
N1	0.0563 (13)	0.0520 (13)	0.0512 (12)	0.0260 (10)	−0.0003 (10)	−0.0015 (10)
O1	0.0468 (10)	0.0774 (13)	0.0507 (11)	0.0283 (9)	0.0027 (8)	0.0039 (9)
O2	0.0901 (16)	0.0779 (14)	0.0548 (12)	0.0428 (12)	−0.0140 (11)	−0.0167 (11)
O3	0.0637 (12)	0.0609 (12)	0.0513 (11)	0.0312 (10)	−0.0071 (9)	−0.0008 (9)
O4	0.0549 (12)	0.0869 (15)	0.0590 (12)	0.0379 (11)	0.0086 (9)	0.0100 (10)
C1	0.0461 (14)	0.0585 (15)	0.0465 (14)	0.0220 (12)	0.0049 (11)	0.0049 (12)
C2	0.0600 (16)	0.0603 (16)	0.0524 (15)	0.0355 (14)	0.0043 (13)	0.0020 (13)
C3	0.0634 (17)	0.0516 (15)	0.0470 (14)	0.0295 (13)	0.0039 (12)	−0.0020 (11)
C4	0.0654 (17)	0.0523 (15)	0.0507 (15)	0.0339 (13)	0.0019 (13)	0.0007 (12)
C5	0.0665 (17)	0.0570 (16)	0.0468 (14)	0.0299 (14)	−0.0005 (13)	−0.0061 (12)
C6	0.0512 (14)	0.0463 (13)	0.0450 (13)	0.0219 (11)	0.0058 (11)	0.0030 (11)
C7	0.0563 (15)	0.0482 (14)	0.0442 (13)	0.0220 (12)	0.0046 (11)	0.0001 (11)
C8	0.0502 (14)	0.0469 (13)	0.0429 (13)	0.0206 (11)	0.0040 (11)	0.0026 (10)
C9	0.0581 (16)	0.0522 (15)	0.0493 (14)	0.0249 (13)	0.0031 (12)	0.0025 (12)
C10	0.0603 (10)	0.0662 (11)	0.0616 (10)	0.0281 (8)	−0.0028 (8)	0.0013 (8)
C11	0.0603 (10)	0.0662 (11)	0.0616 (10)	0.0281 (8)	−0.0028 (8)	0.0013 (8)
C12	0.0472 (15)	0.0487 (14)	0.0487 (14)	0.0173 (12)	−0.0005 (11)	−0.0029 (11)
C13	0.0603 (10)	0.0662 (11)	0.0616 (10)	0.0281 (8)	−0.0028 (8)	0.0013 (8)

### Geometric parameters (Å, °)

**Table:**

N1—C10	1.275 (3)	C4—C6	1.400 (3)
N1—C8	1.409 (3)	C4—H4	0.9300
O1—C12	1.363 (3)	C5—H5	0.9300
O1—C1	1.405 (3)	C6—C7	1.450 (3)
O2—C9	1.196 (3)	C7—C8	1.345 (4)
O3—C10	1.381 (3)	C7—H7	0.9300
O3—C9	1.390 (3)	C8—C9	1.463 (4)
O4—C12	1.185 (3)	C10—C11	1.469 (4)
C1—C2	1.374 (4)	C11—H11A	0.9600
C1—C5	1.376 (4)	C11—H11B	0.9600
C2—C3	1.379 (4)	C11—H11C	0.9600
C2—H2	0.9300	C12—C13	1.484 (4)
C3—C6	1.398 (3)	C13—H13A	0.9600
C3—H3	0.9300	C13—H13B	0.9600
C4—C5	1.375 (4)	C13—H13C	0.9600
			
C10—N1—C8	105.4 (2)	C7—C8—N1	129.4 (2)
C12—O1—C1	118.8 (2)	C7—C8—C9	122.6 (2)
C10—O3—C9	105.4 (2)	N1—C8—C9	108.0 (2)
C2—C1—C5	121.3 (2)	O2—C9—O3	121.5 (2)
C2—C1—O1	116.6 (2)	O2—C9—C8	133.5 (2)
C5—C1—O1	122.0 (2)	O3—C9—C8	105.0 (2)
C1—C2—C3	119.3 (2)	N1—C10—O3	116.3 (2)
C1—C2—H2	120.3	N1—C10—C11	128.7 (3)
C3—C2—H2	120.3	O3—C10—C11	115.1 (2)
C2—C3—C6	121.1 (2)	C10—C11—H11A	109.5
C2—C3—H3	119.5	C10—C11—H11B	109.5
C6—C3—H3	119.5	H11A—C11—H11B	109.5
C5—C4—C6	121.1 (2)	C10—C11—H11C	109.5
C5—C4—H4	119.4	H11A—C11—H11C	109.5
C6—C4—H4	119.4	H11B—C11—H11C	109.5
C4—C5—C1	119.4 (2)	O4—C12—O1	123.0 (2)
C4—C5—H5	120.3	O4—C12—C13	125.9 (3)
C1—C5—H5	120.3	O1—C12—C13	111.1 (2)
C3—C6—C4	117.8 (2)	C12—C13—H13A	109.5
C3—C6—C7	118.6 (2)	C12—C13—H13B	109.5
C4—C6—C7	123.6 (2)	H13A—C13—H13B	109.5
C8—C7—C6	130.1 (2)	C12—C13—H13C	109.5
C8—C7—H7	114.9	H13A—C13—H13C	109.5
C6—C7—H7	114.9	H13B—C13—H13C	109.5

### Hydrogen-bond geometry (Å, °)

**Table:**

*D*—H···*A*	*D*—H	H···*A*	*D*···*A*	*D*—H···*A*
C4—H4···N1	0.93	2.45	3.098 (4)	127
C7—H7···O2^i^	0.93	2.53	3.417 (4)	160
C13—H13B···O4^ii^	0.96	2.50	3.382 (4)	152

Symmetry codes:  (i) −*x*+1, −*y*+1, −*z*; (ii) *x*+1, *y*, *z*.
